# Ready-Made Oral Nutritional Supplements Improve Nutritional Outcomes and Reduce Health Care Use—A Randomised Trial in Older Malnourished People in Primary Care

**DOI:** 10.3390/nu12020517

**Published:** 2020-02-18

**Authors:** Trevor R. Smith, Abbie L. Cawood, Emily R. Walters, Natasha Guildford, Rebecca J. Stratton

**Affiliations:** 1 Department of Gastroenterology, Mailpoint 255, University Hospitals Southampton NHS Foundation Trust, Southampton General Hospital, Tremona Road, Southampton SO16 6YD, UK; 2 Institute of Human Nutrition, Faculty of Medicine, Mailpoint 113, Southampton General Hospital, Tremona Road, Southampton SO16 6YD, UK; A.L.Cawood@soton.ac.uk (A.L.C.); R.J.Stratton@soton.ac.uk (R.J.S.); 3 Faculty of Health Sciences, University of Southampton, University Road, Southampton SO17 1BJ, UK; erw2v07@soton.ac.uk; 4 Department of Nutrition and Dietetics, University Hospital Southampton NHS Foundation Trust, Southampton General Hospital, Tremona Road, Southampton SO16 6YD, UK; Natasha.Guildford@uhs.nhs.uk

**Keywords:** malnutrition, oral nutritional supplement, dietary advice, health care use, free living elderly

## Abstract

Large trials assessing oral nutritional supplements (ONS) and dietary advice (DA) in primary care are lacking. This study examined effects of ONS + DA versus DA on intake, weight, QoL, healthcare use and satisfaction in malnourished free-living older people. Three hundred and eight people (71.5 ± 10.7y) were randomised to receive ONS + DA (n154) or DA (n154) for 12 weeks. At baseline, 4, 8, 12 weeks, intake, weight, QoL, healthcare use and satisfaction were measured. ONS + DA group (mean daily intake ONS 480 kcal; 21 g protein; 80% compliance) had significantly greater total energy and protein intakes (+401 kcal/d, *p* < 0.001; +15 g/d, *p* < 0.001) and weight gain (+0.8 kg; *p* < 0.001) compared to DA. QoL improved in both groups over time with a significant improvement in index with ONS + DA (*p* = 0.009). Significantly more participants found ONS + DA made a difference for them (*p* = 0.011), but no differences were found between groups using Euroqol. Compared to DA, healthcare use reduced with ONS + DA, (HCP visits by 34%, emergency admissions 50%, LOS 62%). Acceptability of both interventions was high (ONS 96%, DA 95%), with significantly more participants satisfied with ONS (89%) than DA (73%) (*p* = 0.009). This trial in primary care indicates that ONS are acceptable, make a difference to patients, significantly improve intake and weight, and reduce health care use with potential savings.

## 1. Introduction

Disease-related malnutrition (DRM), is a common problem that adversely affects body form and function [1].This often results in lack of energy, more disease complications, slower recovery from illness, and impaired health-related quality of life (QoL) [1]. These patient outcomes place increased clinical and economic demands on primary and secondary care [2]. In the UK the overall cost of malnutrition (disease being the most common cause, DRM), is estimated to be around 15% of the total public expenditure on health and social care (£23.5 billion in the UK) [2]. This economic report highlights the importance of identifying and managing malnutrition as the health and social care costs of a malnourished person are 3–4 times greater each year compared to a non-malnourished person [2]. It is particularly important to focus efforts to manage malnutrition in the community (primary care) as the vast majority of people who are malnourished reside outside of hospital in this setting (~93%) [3]. If it is not effectively treated in the primary care setting, DRM can extend to other settings, for example around 30% of adults admitted to UK hospitals are malnourished or at risk of malnutrition [4].

Nutritional interventions for DRM are known to produce clinical and economic benefits [1,2,5,6,7,8], but there is surprisingly little information specifically in the primary care setting [9] where people initially consult their GP (general practitioner) for advice. The lack of clinical trials in primary care may be due to organisational difficulties of running trials in this setting as potential recruits may be hard to identify and also be widely spread across the local area. Typically, there is limited dietetic resource in primary care, so other health care professionals with variable training in nutrition often manage malnutrition. Nevertheless, current recommended first line treatments include dietary advice (DA), often given as a written information sheet, and oral nutritional supplements (ONS) [9]. Ready-made liquid ONS have been shown to have both clinical and economic benefits in a variety of patient groups (including COPD, frailty, gastrointestinal conditions, wounds, neurological conditions, cancer and those recently discharged from hospital) [8] in both hospitals and care homes [7,10,11,12], but evidence in free living people remains limited. As far as we are aware few, if any, large randomised trials in primary care have been undertaken of first line nutritional interventions such as DA and ONS. This is despite the guidance provided by the National Institute for Health and Care Excellence (NICE), and their calculations of the substantial cost savings made by managing malnutrition [13]. NICE have recommended the need to establish an evidence base for the use of ONS and other nutritional interventions in the community [9]. Therefore, this pragmatic randomised trial was undertaken to investigate: (i) Whether first line treatment for malnutrition using a combination of ONS and DA is more effective than DA alone, at improving nutritional outcomes, QoL, healthcare use and (ii) the acceptability and satisfaction of both ONS and DA interventions, in free living older malnourished people.

## 2. Materials and Methods

### 2.1. Trial Design

This was a prospective, randomised, parallel, open-label trial, which took place between December 2012 and April 2016, across 7 counties in England, UK (Dorset, Hampshire, Wiltshire, Surrey, Sussex, Somerset, Gloucester). Three hundred and eight free living malnourished older people were recruited from general practice and randomised to receive either ready-made energy dense ONS (2.4 kcal/mL) plus DA provided as a diet sheet or DA alone for a period of 12 weeks.

### 2.2. Selection of Participants

Individuals were eligible to participate if they were aged >50 years, were at medium or high risk of malnutrition (according to the Malnutrition Universal Screening Tool (MUST), described later), if the malnutrition was disease related, and they were able to eat and drink and provide informed consent. Exclusion criteria included galactosaemia or known lactose intolerance (contraindications for ONS), those already receiving any form of nutritional support, chronic renal disease requiring dialysis, dysphagia, poorly controlled diabetes, liver failure, cancer (active malignancy), end of life care, residing in an institution, participation in other clinical trials, unable to provide informed consent (e.g., dementia) and those not at risk of DRM.

Screening for potential participants against the inclusion and exclusion criteria was undertaken in two phases, phase 1 via database searches using read codes at GP surgeries and phase 2 by the research team over the phone initially and then in person if they were potentially eligible.

**Figure 1:**
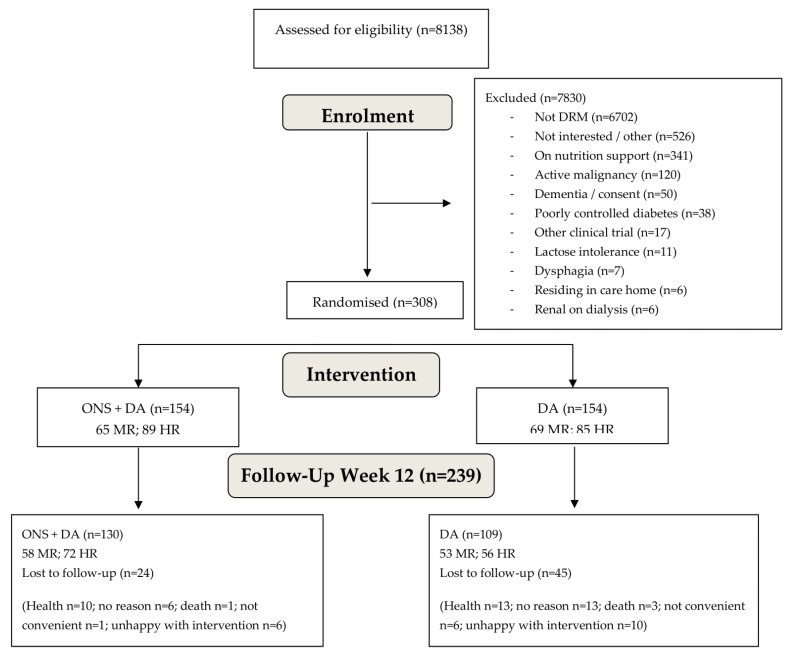
Flow diagram of participants from screening to week 12 follow up visit. ONS, oral nutritional supplement. DA, dietary advice. MR, medium risk. HR, high risk.

GP surgeries, acting as participant information centres, were approached either by the research team directly or via the National Institute of Health Research Primary Care Research Network teams (NIHR PCRN). Those interested in taking part were visited by a member of the research team and provided with an information folder containing all relevant information regarding the trial. GP surgeries ran searches to include individuals 50 years or older, and exclude palliative care, dementia, chronic renal impairment (level 3–5), and poorly controlled diabetes. Following patient database searches undertaken by the surgeries, surgeries used a confidential and secure electronic mail system (Docmail, CFH Docmail Ltd., Radstock, UK) to send a standard letter to potential participants who were invited to contact the research team (via email, freephone number or freepost envelope). In total 332,024 letters were sent from 179 GP practices, and 8905 people contacted the research team. Of these 8138 participated in a screening telephone call undertaken by the research team (767 could not be contacted, despite attempts being made on three separate occasions). The research team asked the individuals to self-report height, weight and previous weight, so that they could establish a malnutrition risk category based on MUST [14] which includes BMI (step 1 of MUST) and percentage unintentional weight loss (step 2 of MUST). If the individual was at risk of malnutrition further questions were asked about weight, illness and disease, current prescription of any type of nutrition support and participation in other clinical trials. If the individual was potentially eligible at this stage, a home visit was booked, and the full screening procedure was undertaken. In total 615 individuals had a screening visit at their home, and of these 308 were enrolled in the study after providing informed consent (Figure 1). The reasons for ineligibility included; those not at risk of DRM (*n* = 6702), not interested in participating (*n* = 492), receiving ONS (*n* = 197), receiving dietary advice (*n* = 141), cancer (active malignancy or recent active malignancy) (*n* = 120), dementia or concerns regarding consent (*n* = 50), poorly controlled diabetes (*n* = 38), not giving a reason for not wanting to take part (other reasons) (*n* = 34), participation in another trial (*n* = 17), lactose intolerance/galactosaemia (*n* = 11), dysphagia (*n* = 7), chronic renal failure requiring dialysis (*n* = 6), in residential care home (*n* = 6), liver failure (*n* = 4) on any form of tube feeding (*n* = 3), palliative care (*n* = 2) (Figure 1).

### 2.3. Randomisation Procedure

Participants were randomised into one of two intervention groups using computer generated random number tables with stratification for malnutrition risk (medium or high risk). Randomisation lists were generated, using a computer programme, by the chief investigator and research fellow prior to commencement of the trial, the details of which were unknown by the research dietitian undertaking the home visit. At the end of the baseline visit the research dietitian enrolled participants and assigned interventions by contacting the research assistant based at the office who provided the randomisation code after opening the next sealed opaque envelope containing the designated intervention.

### 2.4. Interventions

The research dietitian provided all nutritional interventions, in person at the participants own home. Participants received either DA alone, in the form of verbal instructions on the modification of food intake (i.e., food fortification, addition of snacks) and were given a widely available diet sheet to refer to (“Eating well with a small appetite”, NAGE, Older People Specialist Group, British Dietetic Association) or the same DA plus ready-made energy dense liquid ONS, high in energy and protein in a range of seven flavours ad libitum (125 mL containing 300 kcal, 12–18 g protein, vitamins and minerals, Fortisip Compact range, Nutricia, Trowbridge, Wiltshire, UK). If a participant did not like milk (n9), they were offered a non-milk based ONS, also in a range of seven flavours (200 mL containing 300 kcal, 8 g protein, vitamins and minerals, Fortijuce, Nutricia). ONS were delivered to the participant’s home by the research dietitian. The research dietitian undertook a taste test for those in the ONS group so that taste preferences could be taken into account. Verbal instructions and an information sheet about ONS were provided. Although intake was voluntary, participants were asked to aim to consume at least 600 kcal and 16 g protein per day (250–400 mL/day). Participants were advised to drink the ONS for 12 weeks and remained in the trial irrespective of the quantity of the ONS consumed.

### 2.5. Measurements and Outcomes

Age, gender, medical history, Charleston co-morbidity index (CCI) and height (to the nearest 1 cm using a Leicester Height Measure) were recorded at the baseline visit only. At each time point all other measures were undertaken. Body weight was measured to the nearest 0.1 kg using Marsden (MS-4202L) calibrated scales, BMI (weight/height^2^) (step 1 of MUST), percentage unintentional weight loss (step 2 of MUST), MUST score and overall risk category (medium or high risk) were calculated.

The primary outcome measure was QoL and the secondary outcomes were health care use (HCP visits including those to the GP, hospital admissions and length of hospital stay), mortality, dietary intake, weight, compliance and satisfaction. All data were collected in participant’s own homes at baseline, week 4 (±2 days), week 8 (±2 days) and week 12 (±2 days). QoL was assessed using the Euroqol EQ-5D-5L tool [15,16], which consists of a descriptive system of five domains (mobility, self-care, usual activities, pain/discomfort, anxiety/depression) each having five levels (no problems, slight problems, moderate problems, severe problems, extreme problems). The participant was asked to indicate their health state by ticking the box next to the most appropriate statement for each of the five dimensions so that each dimension could be coded into a one-digit number. The digits for the five dimensions were then combined to establish the participant’s overall QoL index (health state range −0.281 to 1.00). QoL was also established using the Euroqol Visual Analogue Scale (VAS) ranging from 0 to 100, with 0 representing the ‘worst health you can imagine’ and 100 the ‘best health you can imagine’. In addition, all participants answered a subjective quality of life question “Has the intervention made a difference to you?” Healthcare use (all healthcare professional appointments, hospital admissions and length of hospital stay) was recorded by the participants in diaries and checked by the research dietitian at each planned visit. If the participant had not completed this in advance of their baseline visit, the research dietitian asked them to complete it during the visit. The number needed to treat was given by the reciprocal of the absolute risk reductions [17]. Dietary intake was assessed by a dietitian at baseline using the 24-h diet recall method and at weeks 4, 8 and 12 using a 3-day diet record. The 3-day diet record was completed by the participant in the 3 days prior to each home visit. If the participant did not complete the diet record, the dietitian completed a 24-h recall. Both methods included the intake of all foods, drinks and ONS. A checklist was used to ensure all relevant items were considered and diet records were reviewed by the research dietitian with the participant to clarify any queries. Intakes of energy and protein from the diet alone, from ONS and total intake were analysed (WISP version 4, Tinuviel, Anglesey UK). Compliance to ONS, also assessed by the research dietitian, was taken to be the amount of ONS consumed as a proportion of that advised (600 kcal). Compliance to DA was assessed by asking participants whether they had made any dietary changes and providing a yes/no answer. Satisfaction, convenience and acceptability for ONS and DA was assessed by a series of questions asked by the research dietitian, which included three “yes/no” questions; “Did you find the intervention convenient?”, “Did you find the intervention acceptable?”, “Did you find the DA easy to follow?”, and one satisfaction question “What was your overall satisfaction with the intervention?”, assessed using a 5-point scale, with 5 being ‘most satisfied’ and 1 ‘least satisfied’.

### 2.6. Ethics

Ethical approval from the Southampton Central Southampton Research Ethics Committee A was granted in September 2012 and the trial was conducted in accordance with their ethical standards and the Declaration of Helsinki. The trial was registered with ISRCTN database on 3^rd^ October 2012; registration number ISRCTN26004104. The trial was sponsored by University Hospital Southampton NHS Foundation trust and was adopted by the National Institute of Health Research (NIHR). All ‘serious adverse events’ were logged and reported to the research ethics committee. Participants received no inducement for taking part in the trial, including travel as they were visited in their own home.

### 2.7. Statistics and Data Analysis

Sample size calculations for the primary outcome (QoL) were undertaken using both the QoL index (range −0.281 to 1) and VAS (range 0–100). It was assumed that the SD of the EQ-5D-5L index was 0.27 for a mixed group of individuals suffering from a variety of conditions [18], and that the correlation between the baseline and post-intervention values was 0.75, in line with observations from our previous randomised clinical trials involving ONS and DA. A sample size of 150 per group was considered to be sufficiently large to detect within subject changes that differed between groups by 0.062, a clinically relevant difference, with 80% power and a *p* value of 0.05. Using different assumptions, before normative values for EQ-5D-5L became available for large samples of subjects suffering from a wide range of clinical conditions, it was calculated that a sample size of 150 per group would be sufficient to detect a difference in VAS of 7.5 between groups (SD of the change within group 23; correlation 0.5) with the same power and *p* value.

Two types of analyses were undertaken to compare the results obtained in the DA group with those of the ONS + DA group: An intention to treat analysis (ITT), and a per protocol (PP) analysis for those with complete datasets at 4 weeks, 8 weeks and 12 weeks. Primary and secondary outcome data without adjustment for covariates or confounding variables were analysed using Chi squared tests (categorical variables), and both paired and unpaired t-tests (continuous variables). Analyses involving adjustment for covariates or confounding variables, baseline values, MUST category, age, gender, and CCI were also undertaken using binary logistic regression and the General Linear Model (Univariate Analysis of Variance). Intention to treat analysis was undertaken following multiple imputation [19] involving 5 sets of imputed data. Pooling of data was undertaken using Rubin’s rules [20] and the associated degrees of freedom and *p* values were calculated using the methodology recommended by Barnard and Rubin [21] which is regardless of sample size and can be applied both to small and large sample sizes. All the analyses and imputations were undertaken using SPSS version 22.0 (Chicago). All statistical analyses were undertaken by an independent statistician not involved in the clinical trial, blinded to group allocation. Results are presented as proportions (for categorical variables), odds ratios (binary outcome variables) and mean ± SD (or mean ± SE) for analyses involving modelling using the General Linear Model (Univariate Analysis of Variance) (continuous variables) or odds ratio (binary outcome variables). All data were adjusted for baseline values, MUST category, age, gender, and CCI unless otherwise stated.

## 3. Results

### 3.1. Recruitment and Baseline Characteristics of Participants

308 free living older people (mean ± SD age 71.47 ± 10.74 years; BMI 19.40 ± 2.49 kg/m^2^; 67% female; 44% medium risk and 56% high risk according to MUST) were recruited between December 2012 and January 2016, with week 12 follow up visits completed by April 2016. 154 participants were randomised to receive ONS plus DA and 154 to receive DA only (Table 1; Figure 1). The trial ended when the target recruitment was reached, and all participants had completed their follow up visits. The main health problems for the group were respiratory (34%, including COPD which accounted for 75% of this group), gastrointestinal (29%, including ulcerative colitis, Crohn’s disease), and musculoskeletal (13%, including arthritis, fractures and osteoporosis). No significant differences were seen between the groups at baseline for gender, age, weight, height, BMI, CCI, energy and protein intake or health problems (Table 1).

### 3.2. Dropouts and SAE

From the whole group, 69 subjects (22.4%) dropped out of the study by 12 weeks, the main reasons being participants health (33.4%), unhappy with intervention (23.2%), no reason given (27.5%), not convenient to visit (10.1%) (Figure 1). The baseline subject characteristics (age, gender, CCI, MUST category) of those who dropped out did not differ significantly from those who completed the study, apart from QoL which was significantly lower in those who dropped out (mean EQ-5D index 0.663 v 0.756; *p* = 0.008; mean EQ-5D VAS 59.1 v 66.7, *p* = 0.004).

When considering each group separately, there were significantly fewer dropouts from the ONS + DA group than the DA group at all time points (Table 2). When subdivided by type of intervention there were no significant differences in any of the characteristics at baseline including QoL. Similarly, when those who completed the 12-week intervention period were subdivided by type of intervention, none of the characteristics differed significantly between groups at baseline (e.g., EQ-5D index 0.747 (DA) v 0.762 (ONS + DA), *p* = 0.648; EQ-D VAS 65.9 v 67.5, *p* = 0.499).

There were 46 SAE’s in the DA group and 31 in the ONS + DA group (*p* = 0.722). The number of participants with one or more SAE was not different between the groups (DA n30; ONS + DA n24, *p* = 0.383). All SAE’s were rated “not likely” or “unlikely” to be related to the study product.

### 3.3. Quality of Life (QoL)

#### 3.3.1. Comparison with Normative Values and Changes within Groups

The mean baseline EQ-5D 5L index, 0.735 (SD 0.232) (*n* = 308) for this group of individuals at medium and high risk of malnutrition, was similar to that estimated for the general population in England once adjusted for age (0.021 units below; 2.8%) [22].The baseline EQ-5D VAS, 65.0 (SD 19.28), was 15% (10.9 units) below that estimated for the general population [22]. The correlation coefficient between baseline EQ-5D 5L index and EQ-5D VAS was only 0.632 (*r*^2^ = 0.400).

During the 12-week intervention period, for both groups combined, the mean values for EQ-5D VAS (mean ± SE) were found to be significantly higher than the baseline values by 1.9 ± 0.9 units (corresponding to 2.9% ± 1.4% of the baseline value) using ITT analysis (*p* = 0.045), and by 3.4 ± 0.7 units (5.0% ± 1.1%) using the PP analysis (*p* = 0.001). The improvements obtained with EQ-5D VAS were greater than those with the EQ-5D-5L index in the same subjects (by 1.3% ± 1.5%, *p* = 0.298 using the ITT analysis, and by 2.23% ± 1.09%, *p* = 0.042 using the PP analysis).

There were also improvements within intervention groups. During the 12 weeks the EQ-5D VAS average value rose significantly above the baseline values in the ONS + DA group in both ITT and PP analysis, but only in the PP analysis of the DA group. With the EQ-5D-5L index, values at 12 weeks were universally higher than the baseline values, with the only significant improvement seen in the ONS+ DA group (increase of 0.022 ± 0.008; *p* = 0.009; PP analysis).

#### 3.3.2. Differences between Groups

There were no significant baseline differences between the groups for QoL including VAS and combined index scores (total group mean ± SD 0.74 ± 0.23 index; 65.03 ± 19.28 VAS). There were also no differences in the domains at baseline except for ‘anxiety/depression’ which was slightly worse in the DA group; *p* = 0.043 (data not shown).

There were no significant differences in QoL between groups at any time point during the 12-week intervention period, and no significant differences in the average values (Table 3). There were also no significant differences between groups in the specific QoL domains. When participants were asked “Has the intervention made a difference to you?” their answer significantly favoured the ONS + DA group (70–75% answered yes at 4, 8 and 12 weeks in ONS + DA group v 50–62% in DA group, with *p*-values ranging from 0.004 to 0.045). Overall at 12 weeks more participants in the ONS + DA (75%) reported the intervention made a difference to them compared to DA (61%) which was significant between groups in both ITT and PP analysis (OR 2.256; 95% CI 1.218–4.176; *p* = 0.011 ITT; OR 2.014; 95% CI 1.131–3.584; *p* = 0.017 PP)

### 3.4. Healthcare Use and Mortality

There were no significant baseline differences between the groups in health care use (apart from length of stay which was longer in the DA group (1.75 ± 1.50 days ONS + DA v 6.67 ± 2.89 days DA; *p* = 0.031). During the 12-week intervention period the various types of healthcare use investigated were universally lower in the ONS + DA group versus DA (Table 4) and were significantly so for some. There were significantly fewer HCP visits (including those to the GP) in the ONS + DA group compared to DA alone according to the ITT analysis (5.1 ± 0.7 v 7.7 ± 1.0; *p* = 0.010), although this was not significant in the PP analysis (Table 4). On average over the initial 4 and 8-week intervention period for both analyses (ITT and PP) there were significantly fewer HCP visits in the ONS + DA group compared to DA group (0–4 weeks 1.5 ± 0.2 ONS + DA v 2.1 ± 0.2 DA, *p* = 0.02 (ITT); 0–8 weeks 3.2 ± 0.4 ONS + DA v 4.9 ± 0.5 DA, *p* = 0.01 (ITT)).

Over 12 weeks a total of 19 participants had 21 admissions, 9 admissions in the ONS + DA group and 12 in the DA group, with 2 participants in the DA group also having a readmission. The number needed to treat based on admissions was 24. Total hospital admissions (emergency plus elective) were less with ONS + DA compared to DA alone (42% less between groups ITT, *p* = 0.298; 64% less PP, *p* = 0.060) although not significant (Table 4). More specifically those who received ONS + DA had fewer emergency hospital admissions compared to DA, which was significant in the PP analysis (50% less between groups ITT, *p* = 0.202; 73% less PP, *p* = 0.026).

The mean length of hospital stay was also shorter in the ONS + DA arm compared to DA according to both the ITT analysis (0.67 ± 0.49 days v 1.78 ± 0.81 days; *p* = 0.161), reaching significance in the PP analysis (0.15 ± 0.13 v 0.57 ± 0.15; *p* = 0.031) (Table 4). There were no significant differences in mortality between the two groups over the 12-week period (3 deaths DA group; 1 death ONS + DA group).

### 3.5. Dietary Intake, Weight and Malnutrition Risk

#### 3.5.1. Dietary Intake

**Figure 2:**
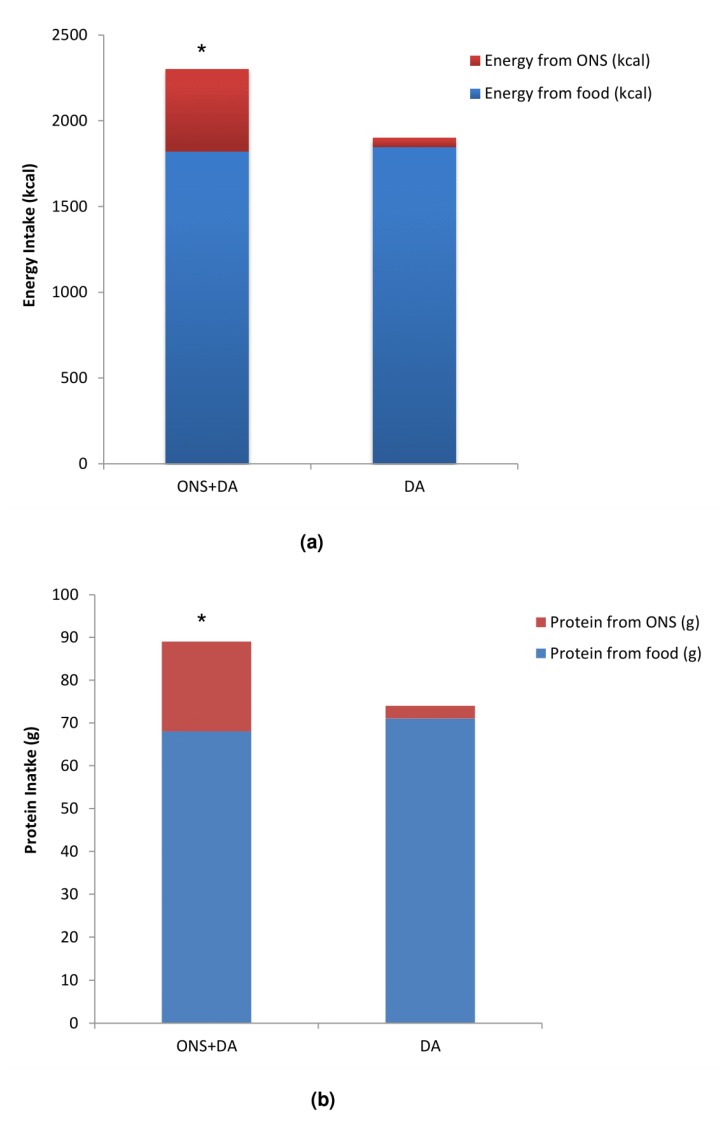
(**a**) Mean daily energy intakes according to intervention group; (**b**) Mean daily protein intakes according to intervention group; Univariate analysis adjusted for the baseline value, MUST category, CCI, age and gender. Data presented as mean ± SE, * *p* < 0.001. 4 participants in the DA arm reported taking over the counter ONS contributing to their intake. DA, dietary advice. ONS, oral nutritional supplement.

Intake of energy and protein did not differ between groups at baseline (total group mean ± SD 1735 ± 564 kcal/d, 65 ± 22 g/d). Over the 12 weeks, total energy and protein intakes were significantly higher in the ONS + DA group than the DA group alone according to ITT analysis (+401 kcal; +15 g protein; *p* < 0.001), and PP analysis (+436 kcal; +19 g protein; *p* < 0.001) (Figure 2). There were no significant differences between groups in food intake in either the ITT (1820 ± 41 kcal/d, 68 ± 1.6 g protein/d ONS + DA vs.1848 ± 43 kcal/d, 71 ± 1.7 g protein/d DA, mean ± SE; *p* > 0.05) or PP analysis (1848 ± 43 kcal/d, 68 ± 1.4 g protein/d ONS + DA vs.1867 ± 44 kcal/d, 71 ± 1.6 g protein/d DA, mean ± SE; *p* > 0.05). The mean daily intake of ONS in the ONS + DA group over the 12-week period was 480 ± 12 kcal, 21 ± 0.6 g protein in the ITT analysis, and 497 ± 12 kcal and 22 ± 0.7 g protein in the PP analysis.

#### 3.5.2. Weight and Malnutrition Risk

Body weight did not differ between the groups at baseline (for both groups combined mean ± SD 52.12 ± 9.52 kg) but following intervention weight was significantly higher at all time points in the ONS + DA group compared to the DA group. On average over the 12 weeks, after adjusting for confounding variables (described in statistics and data analysis), weight was significantly greater (+0.8 kg; *p* < 0.001) in the ONS + DA group compared to the DA group in both the ITT (53.3 ± 0.16 ONS + DA v 52.5 ± 0.2 DA, *p* = 0.003) and PP analysis (53.0 ± 0.17 ONS + DA v 52.2 ± 0.2 DA, *p* = 0.001). Body weight increased significantly from baseline to the end of the 12-week intervention period in the ONS + DA group (+1.5 kg ITT; +1.4 kg PP analysis), but not in the DA group (+0.4 kg ITT and PP analysis). Consequently, the proportion of individuals whose risk of malnutrition was reduced by the end of the intervention, was greater in the ONS + DA group ((55%) had a lower risk of malnutrition at week 12 compared to baseline) compared to the DA group alone (37%) (*p* = 0.021).

### 3.6. Satisfaction and Compliance

Acceptability to both interventions was high, with 92–96% of participants finding them acceptable at weeks 4, 8 and 12, (ONS + DA 96%; DA 95% at week 12). Most participants also found the DA easy to follow (92%). There were no significant differences between groups for either question at any time point. Compared to DA, more participants taking ONS found the intervention convenient, which was significant in the ITT analysis (97% v 92% at week 12; OR 1.908; 95%CI 1.042–3.496; *p* = 0.036 ITT).

When asked if they were “satisfied with the intervention” the answers significantly favoured the ONS + DA group (89–92% satisfied ONS + DA versus 73–77% DA at weeks 4, 8 and 12, with *p* values ranging from 0.001 to 0.002). When the answers were adjusted for confounding variables using binary logistic regression, they also significantly favoured the ONS + DA group over the entire 12 weeks (OR 3.351 ± 0.091 ONS + DA v 2.908 ± 0.130 DA, *p* < 0.001 ITT).

Compliance to ONS (amount consumed as a proportion of that prescribed) was found to be 80% in the ITT analysis and 83% in the PP analysis. For compliance to DA 66 ± 8.8% reported making dietary changes during the intervention period in the DA group, compared to 43 ± 9.9% in the ONS + DA group.

## 4. Discussion

This is the first large randomised trial of malnourished older people in primary care to show that the combination of ready-made energy dense ONS and DA are highly acceptable and more effective than DA alone at increasing intake and weight and reducing the use of healthcare resources and malnutrition risk, whilst making a difference for patients.

Improvements in QoL did not differ significantly between groups, which could be either because both interventions were effective (or ineffective) or because EQ-5D-5L was not sensitive enough. Large scale studies involving the “Patient Reported Outcome Measures” [23], used by the National Health Service in England, show that patient-administered disease-specific QoL tools [24,25,26] are more sensitive than EQ-5D. Disease-specific tools were not used in this study, partly because they are not available for the large variety of diseases, and partly because existing tools have different scales and variable validity. Other explanations for the lack of significant differences in QoL between groups include subjects reporting good QoL before the study began, with little QoL to gain, and the dropout rate being higher than anticipated. Of note participants reporting low or recently declined QoL on study entry were most likely to drop out, further limiting any expected benefit from intervention. This line of reasoning is supported by evidence from studies of different groups undergoing surgery where improvements in QoL were greatest in those with the lowest baseline QoL, and least in those with the highest baseline QoL. In the present study the mean baseline EQ-5D index was estimated to be only 2.8% lower than the age adjusted reference value for the general population of England, implying the potential for improvement in QoL was limited. In contrast, the mean baseline EQ-5D VAS score was 15% lower than the reference value for the general population. This suggests that EQ-5D VAS and EQ-5D index do not measure the same aspects of QoL, explaining why the baseline EQ-5D VAS accounted for only 40% of the baseline variability in the EQ-5D index. Furthermore, ITT analysis of VAS scores showed a significant improvement in both DA and ONS + DA groups whereas EQ-5D index showed no significant improvement in either group. The reason why the baseline EQ-5D index in this study was comparable to that of the general population, despite the presence of both disease and malnutrition is unclear, given reports that a range of different diseases reduce the EQ-5D-5L index below normative population values. Despite the absence of significant differences in QoL between groups, significantly more patients taking ONS rated the intervention as making a difference to them, and being significantly more satisfied, compared to DA alone. It is possible that the satisfaction scores maybe influenced by the patients not having to pay for ONS, as is the case in clinical practice in the UK. Since both groups found the interventions acceptable and easy to follow, the better scores in the ONS + DA group may reflect aspects of QoL not fully captured by EQ-5D. Furthermore, the lower satisfaction scores in the DA group could explain why more subjects dropped out from this group. QoL has been assessed in other studies using ready-made ONS with variable results, some showing improvements in overall scores or domains and other no improvements compared to the control group or other interventions [27,28,29,30,31,32,33,34,35]. This variability may be explained by differences in nutritional status, care setting and baseline QoL scores assessed using different instruments. However, studies showing significant differences between groups have typically favoured the ONS group [27,28,29,30,35,36]. Such studies also reported significant improvements in intake and weight, with reduced health care use.

Healthcare use, ranging from consultations with GPs and visits by HCPs, to number of hospital admissions and length of hospital stays, were universally lower in the ONS + DA than the DA group over the 12-week intervention period, with some being significant (Table 4). A systematic review on use of ONS in the community showed that ONS can significantly reduce hospital admissions or readmissions [10]. However, most of the studies recruited subjects who had just been hospitalised, often commencing ONS in hospital and continuing use in the community, therefore this randomised trial adds to the evidence base. A reduction in health care use with ONS is very positive considering the current economic climate, and supports the evidence base for their cost effectiveness [2,8,11]. Indeed implementation of pathways using ready-made ONS to manage malnutrition in primary care have also shown that the cost of screening and management with ONS are more than offset by reduction in health care use and costs, especially when considering the short period of intervention (12 weeks) [37,38,39,40]. Full economic analyses are required to elucidate the potential cost savings from this dataset.

The mechanism by which ONS reduced health care use is unclear, however it is most likely linked to improved nutritional intake. The ONS intake was responsible for the significantly greater total energy and protein intake and greater weight gain compared to DA alone. The improvement in nutritional status was also reflected in the significantly higher proportion of participants having a reduction in their malnutrition risk in the ONS group. In clinical practice the reduction in malnutrition risk and the resulting improved outcomes may be more important than the increase in body weight alone. The ONS was found to largely add rather than to replace intake from the diet and supports other research indicating that there is little suppression of appetite and food intake with the use of liquid ready-made ONS [7]. Apart from protein and energy, the ONS contained a wide range of nutrients, including micronutrients, which could also be important. The use of low volume (125 mL) high energy density (2.4 kcal/mL) ready-made ONS available in a variety of flavours could have also contributed. Indeed increased energy density is known to aid compliance to ONS, which was excellent in this trial (80–83%), and similar to that reported in a systematic review [41]. It should be noted that this clinical trial replicated current clinical practice in the UK, where patients do not have to pay for ONS as they are foods for special medical purposes and are prescribed by the GP and this could also be a factor in improving outcomes.

It is possible that other forms of oral nutrition support may have similar benefits however there is currently little published data on the clinical or cost-effective outcomes of other types of ONS (e.g., powders) or forms of oral nutrition support. The DA was a set of verbal instructions given by a dietitian, with a corresponding diet sheet left with the participant, this is similar to previously described definitions of DA [6,9,42]. The purpose was to provide simple instruction on modifying food intake (e.g., food fortification, meal plan adaptation) to improve nutritional intake, which could be replicated in primary care, outside of a clinical trial setting. This is not the same as intensive, individualised dietary counselling by a dietitian, which combined with ONS may be the optimal strategy [43]. This approach can however be restricted by limited resources of the dietetic profession, especially in the primary care setting, as such DA was chosen for this study as it represents a pragmatic and viable nutritional first line intervention in this setting.

Although both groups received DA, those randomised to ONS + DA, were significantly less compliant to making dietary changes, reducing exposure to this component of the intervention. Indeed, if the compliance to DA had been similar in both groups those also receiving ONS may have had even greater benefit on outcomes.

Some study limitations need to be acknowledged. Firstly, like most other randomised trials involving ONS, this was an open trial. In addition, the study did not have a control arm receiving either no treatment or routine care. However, providing no treatment may raise ethical difficulties, while providing routine care is likely to be variable across primary care. In the absence of a control group, care should be taken not to conclude that both treatments were ineffective at improving QoL, as both may have been more effective than no intervention or routine care, or both may have prevented a decline in QoL. Secondly, by the end of the intervention period 22.4% of the subjects dropped out of the study. These dropouts had significantly lower QoL scores and were predominantly from the DA group, increasing the risk of bias. However, baseline characteristics did not differ significantly between groups, either among those who remained in the study or those who dropped out and to minimise this risk of bias from dropouts, an ITT analysis was carried out. It is therefore reassuring that the trends obtained by the PP and ITT analyses were consistent between groups: showing no significant difference in Euroqol, and significant differences in dietary intake, weight, satisfaction and aspects of healthcare use, all favouring the ONS + DA group. Finally, this pragmatic trial aimed to replicate clinical care and as such some outcomes of potential interest were not part of data collection, for example other measures of body composition, like muscle mass/function, in addition to weight, may have added further clinical insights to the results. In addition, larger studies, over longer periods of time should be considered in the future.

In summary, although this trial of older malnourished people in primary care found no difference in QoL (Euroqol) between ONS + DA and DA alone, significant improvements in intake and weight with reductions in health care use were seen with ready-made ONS used for 12 weeks and may explain why significantly more people found ONS made a difference to them compared to DA alone.

## Figures and Tables

**Table 1:** Subject characteristics at baseline according to type of intervention.

Characteristics	ONS + DA (n154)		DA (n154)		ALL (n308)		*p* Value *
*Gender*							0.716 ^a^
Male: n	52		49		101		
Female: n	102		105		207		
*MUST Categories*							0.646 ^a^
MUST—Medium risk: n	65		69		134		
MUST—High risk: n	89		85		174		
Age (y): mean ± SD	71.31 ± 11.18		71.63 ± 10.31		71.47 ± 10.74		0.795 ^b^
Height (m): mean ± SD	1.64 ± 0.09		1.64 ± 0.09		1.64 ± 0.09		0.704 ^b^
Weight (kg): mean ± SD	51.94 ± 8.72		52.29 ± 10.28		52.12 ± 9.52		0.749 ^b^
BMI (kg/m^2^): mean ± SD	19.32 ± 2.31		19.49 ± 2.66		19.40 ± 2.49		0.561 ^b^
CCI: mean ± SD	0.94 ± 0.92		1.09 ± 0.94		1.02 ± 0.93		0.159 ^b^
Number of systems ^†^ mean ± SD	2.62 ± 1.22		2.45 ± 1.11		2.54 ± 1.17		0.188 ^b^
Energy intake (kcal): mean ± SD	1759 ± 565		1712 ± 564		1735 ± 564		0.465 ^b^
Protein intake (g): mean ± SD	65.4 ± 21.6		64.0 ± 22.1		64.7 ± 21.8		0.579 ^b^
EQ-5D-5L index: mean ± SD	0.75 ± 0.22		0.72 ± 0.24		0.74 ± 0.23		0.155 ^b^
EQ-5D-5L VAS: mean ± SD	66.54 ± 19.18		63.53 ± 19.03		65.03 ± 19.28		0.172 ^b^
*Health problems*							0.608 ^a^
Respiratory; *n* (%)	54	(35.1%)	52	(33.8%)	106	(34.4%)	
Gastrointestinal; *n* (%)	40	(26.0%)	50	(32.5%)	90	(29.2%)	
Musculoskeletal; *n* (%)	24	(15.6%)	17	(11%)	41	(13.3%)	
Cardiovascular System; *n* (%)	10	(6.5%)	12	(7.8%)	22	(7.1%)	
Central nervous system; *n* (%)	7	(4.5%)	4	(2.6%)	11	(3.6%)	
Other; *n* (%)	19	(12.3%)	19	(12.3%)	38	(12.3%)	

ONS, oral nutritional supplement. DA, dietary advice. CCI, Charleston Comorbidity index. EQ5D, Euroqol. VAS, visual analogue scale. MUST, Malnutrition Universal Screening Tool. ^a^ Chi Squared, ^b^ Independent samples *t*-test. * Relates to comparisons between groups. ^†^ Refers to the number of body systems affected by health problems.

**Table 2:** Analysis of dropouts.

Timepoint	ONS + DA (n154)	DA (n154)	ALL (n308)	*p* Value ^a^	*p* Value ^b^
**Week 4**					
% dropouts	12/154 (7.8%)	27/154 (17.5%)	39/308 (12.7%)	0.010	0.016
Dropouts v no dropouts	12 v 142	27 v 127	39 v 269		
**Week 8**					
% dropouts	19/154 (12.3%)	37/154 (24.0%)	56/308 (18.1%)	0.008	0.006
Dropouts v no dropouts	19 v 135	37 v 117	56 v 252		
**Week 12**					
% dropouts	24/154 (15.6%)	45/154 (29.2%)	69/308 (22.4%)	0.004	0.006
Dropouts v no dropouts	24 v 130	45 v 109	69 v 239		

ONS, oral nutritional supplement. DA, dietary advice; *p* value comparisons between groups, **^a^** Chi squared test (2 tailed); **^b^** Fischer exact test (2 tailed). Data is cumulative.

**Table 3:** Average quality of life (index and VAS) for the 12-week period according to type of intervention and analysis (intention to treat and per protocol).

Quality of Life	Baseline	Average Over Intervention				*p* Value ^a^
		**ONS + DA**		**DA**		
**Intention to treat analysis**						
		***n*** **= 154**		***n*** **= 154**		
EQ-5D-5L index	0.735	0.753	0.011	0.743	0.010	0.425
ED-5D-5L VAS	65.0	67.6	1.1	66.2	1.3	0.344
**Per protocol analysis**						
		***n*** **= 126**		***n*** **= 104**		
EQ-5D-5L index	0.735	0.783	0.009	0.780	0.010	0.815
ED-5D-5L VAS	66.8	69.8	1.0	70.6	1.1	0.582

ONS, oral nutritional supplement. DA, dietary advice. EQ5D, Euroqol. VAS, visual analogue scale; ^a^ Univariate analysis adjusted for the baseline EQ-5D, MUST category, CCI, age and gender; Data presented as mean ± SE.

**Table 4:** Average health care use per subject during the 12-week intervention period according to type of intervention and analysis (intention to treat and per protocol).

Healthcare Use	ONS + DA		DA		*p* Value ^a^
**Intention to treat *(*n*154:*n*154)***					
Total HCP * visits	5.099	0.726	7.676	1.046	0.010
Total hospital admissions	0.070	0.032	0.118	0.033	0.297
Total emergency admissions	0.046	0.028	0.100	0.031	0.202
Total elective admissions	0.025	0.012	0.021	0.013	0.808
Total length of stay (days)	0.670	0.490	1.782	0.807	0.161
**Per protocol** ^†^*(*nONS + DA:nDA*)*					
Total HCP * visits n118:n103	4.139	0.458	4.926	0.525	0.261
Total hospital admissions n128:n108	0.054	0.030	0.140	0.034	0.060
Total emergency admissions n125:n106	0.028	0.025	0.114	0.029	0.026
Total elective admissions n125:n107	0.026	0.013	0.029	0.015	0.877
Total length of stay (days) n126:n107	0.152	0.127	0.572	0.146	0.031

ONS, oral nutritional supplement. DA, dietary advice. Univariate analysis, results (mean ± SE) were adjusted for baseline value of variable being examined, MUST category, CCI, age and gender. ^a^ Independent samples *T* test. * Total HCP visits includes GP visits. ^†^ For subjects who completed the 12 weeks of the study.

## References

[1] Stratton R.J., Green C.J., Elia M. (2003). Disease-Related Malnutrition: An Evidence Based Approach to Treatment.

[2] Elia M. (2015). The cost of malnutrition in England and potential cost savings from nutritional interventions. A Report from the Malnutrition Action Group of BAPEN and the National Institute for Health Research Southampton Biomedical Research Centre.

[3] Elia M., Russell C.A. (2009). Combating Malnutrition: Recommendations for Action.

[4] Russell C.A., Elia M. (2007). Nutrition Screening Surveys in Hospitals in the UK, 2007–2011. In A Report Based on the Amalgamated Data from the Four Nutrition Screening Week Surveys undertaken by BAPEN in.

[5] Cawood A.L., Elia M., Stratton R.J. (2012). Systematic review and meta-analysis of the effects of high protein oral nutritional supplements. Ageing Res. Rev..

[6] Baldwin C., Weekes C.E. (2011). Dietary advice with or without oral nutritional supplements for disease-related malnutrition in adults. Cochrane Database Syst. Rev..

[7] Stratton R.J., Elia M. (2007). A review of reviews: A new look at the evidence for oral nutritional supplements in clinical practice. Clin. Nutr. Suppl..

[8] Stratton R.J., Smith T., Gabe S. Managing Malnutrition to Improve Lives and Save Money.

[9] National Institute for Health and Clinical Excellence (NICE) (2006). Nutrition Support in Adults: Oral Nutrition Support, Enteral Tube Feeding and Parenteral Nutrition (Clinical Guideline 32).

[10] Stratton R.J., Hebuterne X., Elia M. (2013). A systematic review and meta-analysis of the impact of oral nutritional supplements on hospital readmissions. Ageing Res. Rev..

[11] Elia M., Normand C., Laviano A., Norman K. (2016). A systematic review of the cost and cost effectiveness of using standard oral nutritional supplements in community and care home settings. Clin. Nutr..

[12] Elia M., Normand C., Norman K., Laviano A. (2016). A systematic review of the cost and cost effectiveness of using standard oral nutritional supplements in the hospital setting. Clin. Nutr..

[13] National Institute for Health and Clinical Excellence (NICE) (2012). Quality Standard for Nutrition Support in Adults. NICE Quality Standard 24.

[14] BAPEN The ‘MUST’ Toolkit. https://www.bapen.org.uk/screening-and-must/must/must-toolkit.

[15] Herdman M., Gudex C., Lloyd A., Janssen M., Kind P., Parkin D., Bonsel G., Badia X. (2011). Development and preliminary testing of the new five-level version of EQ-5D (EQ-5D-5L). Q. Life Res..

[16] Group T.E. (1990). EuroQol-a new facility for the measurement of health-related quality of life. Health Policy.

[17] Cook R.J., Sackett D.L. (1995). The number needed to treat: A clinically useful measure of treatment effect. BMJ.

[18] Mulhern B., Feng Y., Shah K., Janssen M.F., Herdman M., van Hout B., Devlin N. (2018). Comparing the UK EQ-5D-3L and English EQ-5D-5L Value Sets. Pharmacoeconomics.

[19] Altman D.G. (2009). Missing outcomes in randomized trials: Addressing the dilemma. Open Med.

[20] Rubin D.B. (1987). Multiple Imputation for Nonresponse in Surveys.

[21] Barnard J., Rubin D.B. (1999). Miscellanea. Small-sample degrees of freedom with multiple imputation. Biometrika.

[22] Szende A., Janssen B., Cabases J. (2014). Self-Reported Population Health: An International Perspective Based on EQ-5D.

[23] NHS Digital.Finalised Patient Reported Outcome Measures (PROMs) in England. https://digital.nhs.uk/data-and-information/publications/statistical/patient-reported-outcome-measures-proms/provisional-monthly-patient-reported-outcome-measures-proms-in-england-april-2017-to-june-2017.

[24] Dawson J., Fitzpatrick R., Carr A., Murray D. (1996). Questionnaire on the perceptions of patients about total hip replacement. J. Bone Joint Surg. Br..

[25] Dawson J., Fitzpatrick R., Murray D., Carr A. (1998). Questionnaire on the perceptions of patients about total knee replacement. J. Bone Joint Surg. Br..

[26] Klem T.M., Sybrandy J.E., Wittens C.H. (2009). Measurement of health-related quality of life with the Dutch translated Aberdeen Varicose Vein Questionnaire before and after treatment. Eur. J. Vasc. Endovasc. Surg..

[27] Parsons E.L., Stratton R.J., Cawood A.L., Smith T.R., Elia M. (2017). Oral nutritional supplements in a randomised trial are more effective than dietary advice at improving quality of life in malnourished care home residents. Clin. Nutr..

[28] Krondl M., Coleman P.H., Bradley C.L., Lau D., Ryan N. (1999). Subjectively healthy elderly consuming a liquid nutrition supplement maintained body mass index and improved some nutritional parameters and perceived well being. J. Am. Diet. Assoc..

[29] Gariballa S., Forster S. (2007). Dietary supplementation and quality of life of older patients: A randomized, double-blind, placebo-controlled trial. J. Am. Geriatr. Soc..

[30] Norman K., Kirchner H., Freudenreich M., Ockenga J., Lochs H., Pirlich M. (2008). Three month intervention with protein and energy rich supplements improve muscle function and quality of life in malnourished patients with non-neoplastic gastrointestinal disease—A randomized controlled trial. Clin. Nutr..

[31] Tidermark J., Zethraeus N., Svensson O., Tornkvist H., Ponzer S. (2002). Quality of life related to fracture displacement among elderly patients with femoral neck fractures treated with internal fixation. J. Orthop. Trauma.

[32] Edington J., Barnes R., Bryan F., Dupress E., Frost G., Hickson M., Lancaster J., Mongia S., Smith J., Torrance A. (2004). A prospective randomised controlled trial of nutritional supplementation in malnourished elderly in the community: Clinical and health economic outcomes. Clin. Nutr..

[33] Payette H. (2005). Nutrition as a determinant of functional autonomy and quality of life in aging: A research program. Can. J. Physiol. Pharmacol..

[34] Okabayashi T., Iyoki M., Sugimoto T., Kobayashi M., Hanazaki K. (2011). Oral supplementation with carbohydrate- and branched-chain amino acid-enriched nutrients improves postoperative quality of life in patients undergoing hepatic resection. Amino Acids.

[35] Fouque D., McKenzie J., de Mutsert R., Azar R., Teta D., Plauth M., Cano N. (2008). Use of a renal-specific oral supplement by haemodialysis patients with low protein intake does not increase the need for phosphate binders and may prevent a decline in nutritional status and quality of life. Nephrol. Dial. Trans..

[36] Beattie A.H., Prach A.T., Baxter J.P., Pennington C.R. (2000). A randomised controlled trial evaluating the use of enteral nutritional supplements postoperatively in malnourished surgical patients. Gut.

[37] Brown F., Fry G., Cawood A.L., Stratton R.J. (2019). Economic impact of implementing malnutrition screening and nutritional management in older adults in general practice. J. Nutr. Health Aging.

[38] Baggaley E., Whincup L., Ashman K., Cawood A.L., Davies D., Burns E., Stratton R.J. (2013). Effectiveness of implementing a nurse led policy for the management of malnutrition. Clin. Nutr..

[39] Kominek O., Cawood A.L., Janik L., Evill R., Fitzsimmons B., Webb L., Stratton R.J. (2017). Local implementation of a pathway to manage malnourished COPD patients in the community. Eur. Respir. J..

[40] Cawood A.L., Smith A., Pickles S., Church S., Dalrymple-Smith J., Elia M., Stratton R.J. (2009). Effectiveness of implementing MUST into care homes within Peterborough Primary Care Trust England. Clin. Nutr..

[41] Hubbard G.P., Elia M., Holdoway A., Stratton R.J. (2012). A systematic review of compliance to oral nutritional supplements. Clin. Nutr..

[42] Baldwin C., Weekes C.E. (2008). Dietary advice for illness-related malnutrition in adults. Cochrane Database Syst. Rev..

[43] Reinders I., Volkert D., de Groot L., Beck A.M., Feldblum I., Jobse I., Neelemaat F., de van der Schueren M.A.E., Shahar D.R., Smeets E. (2019). Effectiveness of nutritional interventions in older adults at risk of malnutrition across different health care settings: Pooled analyses of individual participant data from nine randomized controlled trials. Clin. Nutr..

